# Fungal Coculture: Unlocking the Potential for Efficient Bioconversion of Lignocellulosic Biomass

**DOI:** 10.3390/jof11060458

**Published:** 2025-06-17

**Authors:** Rafael Icaro Matos Vieira, Alencar da Silva Peixoto, Antonielle Vieira Monclaro, Carlos André Ornelas Ricart, Edivaldo Ximenes Ferreira Filho, Robert Neil Gerard Miller, Taísa Godoy Gomes

**Affiliations:** 1 Laboratory of Microbiology, Department of Cell Biology, University of Brasília, Brasilia 70910-900, DF, Brazil; rafaelicarom@gmail.com (R.I.M.V.); alencar.peixoto@outlook.com (A.d.S.P.); 2 Center for Microbial Ecology and Technology (CMET), Department of Biotechnology, Faculty of Bioscience Engineering, Ghent University, Coupure Links 653, 9000 Ghent, Belgium; antonielle@gmail.com; 3 Center for Advanced Process Technology and Urban Resource Efficiency (CAPTURE), Frieda Saeysstraat, 9052 Ghent, Belgium; 4 Laboratory of Enzymology, Department of Cell Biology, University of Brasília, Brasilia 70910-900, DF, Brazil; eximenes@unb.br; 5 Laboratory of Protein Chemistry and Biochemistry, Department of Cell Biology, University of Brasília, Brasilia 70910-900, DF, Brazil; ricart@unb.br

**Keywords:** coculture, enzyme production, CAZYmes, metabolites, omics technologies

## Abstract

Microbial decomposition of persistent natural compounds such as phenolic lignin and polysaccharides in plant cell walls plays a crucial role in the global carbon cycle and underpins diverse biotechnological applications. Among microbial decomposers, fungi from the *Ascomycota* and *Basidiomycota* phyla have evolved specialized mechanisms for efficient lignocellulosic biomass degradation, employing extracellular enzymes and synergistic fungal consortia. Fungal coculture, defined as the controlled, axenic cultivation of multiple fungal species or strains in a single culture medium, is a promising strategy for industrial processes. This approach to biomass conversion offers potential for enhancing production of enzymes, biofuels, and other high-value bioproducts, while enabling investigation of ecological dynamics and metabolic pathways relevant to biorefinery operations. Lignocellulosic biomass conversion into fuels, energy, and biochemicals is central to the bioeconomy, integrating advanced biotechnology with sustainable resource use. Recent advancements in -omics technologies, including genomics, transcriptomics, and proteomics, have facilitated detailed analysis of fungal metabolism, uncovering novel secondary metabolites and enzymatic pathways activated under specific growth conditions. This review highlights the potential of fungal coculture systems to advance sustainable biomass conversion in alignment with circular bioeconomy goals.

## 1. Introduction

Intensive exploitation of fossil fuels has significantly altered the global carbon cycle, with carbon reserves accumulated over millions of years released into the atmosphere, primarily in the form of carbon dioxide (CO_2_), intensifying the greenhouse effect and directly contributing to global warming [1]. Greenhouse gas emissions are not limited to the energy sector, with agricultural practices such as deforestation not only reducing the carbon sequestration capacity of forests but also releasing large amounts of stored CO_2_ from biomass and soil. The use of fertilizers and improper management of pastures also increases the emission of methane (CH_4_) and nitrous oxide (N_2_O) gases, with a higher global warming potential than CO_2_ [2].

Scientific evidence points to an alarming scenario. The Paris Agreement aims to limit global warming to 1.5 °C [3], a goal that requires maintaining around 60% of oil and gas reserves and 90% of coal reserves untapped until 2050 [4]. Achieving this target also demands a 3% annual reduction in oil and gas production. By 2023, human activities had already caused an average global temperature increase of 1.49 ± 0.11 °C compared to the pre-industrial period [5]. These findings underscore the critical importance of preserving fossil fuel reserves and implementing aggressive measures to reduce emissions, as essential actions to meet the 1.5 °C target and mitigate the impacts of global warming.

Despite these alarming figures, the transportation sector remains heavily reliant on fossil fuels, consuming approximately 65% of the world’s refined oil [6]. This dependence makes it one of the largest sources of greenhouse gas emissions, highlighting the need for a transition to cleaner energy sources. Biofuels are often seen as a potential solution; however, simply replacing fossil fuels with biofuels is not always appropriate. A successful transition must be both strategic and sustainable, avoiding unintended environmental consequences such as monoculture expansion, biodiversity loss, and competition with food production.

Given the depletion of petroleum reserves and the necessity for measures to reduce carbon emissions, numerous sustainable energy models have been developed as alternatives to fossil fuels. Among these, liquid bioenergy sources, which have been classified into different generations according to raw materials and production processes, offer considerable potential. First-generation (1G) ethanol is primarily produced from food crops, such as sugarcane and maize, where sucrose is converted into ethanol through the action of the yeast *Saccharomyces cerevisiae*. This approach is widely criticized due to its direct competition with food production and the intensive requirement for arable land and water resources, potentially threatening food security. Second-generation (2G) ethanol, in contrast, is produced from lignocellulosic biomass, including agricultural and forestry residues. In this process, polysaccharides present in cellulose and hemicellulose are converted into monosaccharides, which are subsequently fermented into ethanol by microorganisms such as yeasts. For this, enzymes play a crucial role in the initial deconstruction of the plant biomass. More recent advances also include third-generation (3G) ethanol, produced from photosynthetic microorganisms such as algae. This process involves converting algal biomass into ethanol, offering a renewable resource with a lower environmental impact. Finally, fourth-generation (4G) ethanol utilizes metabolic engineering to optimize bioethanol production in genetically modified organisms, such as photoautotrophic microalgae [7].

The microbial decomposition of natural polymers, such as lignin and plant cell wall polysaccharides (cellulose, hemicellulose, and pectin), is fundamental to the global carbon cycle and essential for the development of biotechnological processes in industries [8]. Ascomycete and Basidiomycete fungi play key roles in this process, with secreted extracellular enzymes acting as catalysts for the hydrolysis of cellulose and hemicellulose bonds and releasing stored carbon in the plant biomass [9]. In nature, this mechanism occurs through the action of fungal consortia, where complementary enzyme activities enhance biomass degradation efficiency.

Fungal coculture can be defined as the simultaneous cultivation of different fungal species or strains on a specific substrate under controlled growth conditions. Such cultivation can be effective for exploring interactions between different species, emphasizing enzymes, auxiliary proteins, secondary metabolites, and antimicrobial compounds. Fungal interactions in coculture systems have demonstrated promise in activating metabolic pathways and enhancing the chemical diversity of secondary metabolites. Despite this potential, the role of competition, as opposed to mutualism or cooperation, remains relatively underexplored [10]. Antagonistic interactions between fungi can arise through diverse mechanisms, including the production of antimicrobial metabolites, detoxification or inhibition of toxins, and the expression of virulence factors. These mechanisms constitute integral components of complex microbial defense systems, which in turn determine ecological dynamics and functional outcomes within cocultures. While the use of strains that produce antifungal or cytotoxic compounds may pose a risk of competitive exclusion, such interactions can also offer opportunities to probe a broader spectrum of fungal capabilities. Notably, antagonistic dynamics can serve as stimuli for the activation of silent or differentially expressed biosynthetic gene clusters, facilitating the discovery of novel bioactive molecules [11]. Moreover, secondary metabolites released during coculture may play roles beyond defense, including mediating intra- and interspecific signaling. For instance, in the coculture of *Penicillium fuscum* and *Penicillium camembertii*, unique macromolecules and antibiotic compounds were produced exclusively in coculture, active against pathogens such as *Candida albicans* and *Candida glabrata* [12,13]. Although antagonism may reduce coculture stability, it simultaneously provides a route for uncovering cryptic metabolic pathways and expanding our understanding of fungal functional diversity. A deeper elucidation of these interactions, coupled with strategies to mitigate potential drawbacks, will be essential to harness their full biotechnological potential.

While microbial interactions are typically observed in natural environments, playing an important role in fungal competition and collaboration for limited resources, the application of abundant complex agroindustrial substrates, such as sugarcane bagasse, sawdust, molasses, and residues derived from the processing of soybean and corn, as complex carbon sources in controlled fungal coculture systems is emerging as a promising strategy for consolidation of sustainable biorefinery models for biofuels and other bioproducts from plant biomass. In these systems, participating fungi can secrete efficient enzyme cocktails for holocellulose saccharification that result in fermentable sugars and other high-value products. Holocellulose is the polysaccharide fraction of a biomass (lignocellulose) that includes cellulose and all hemicelluloses after the removal of extractives and lignin. It is a carbohydrate-rich material composed of polymers such as cellulose, hemicellulose, and pectin.

In this context, the development of 2G biofuels, coupled with fungal coculture, represents a promising sustainable strategy for renewable energy production and climate change mitigation. By utilizing agricultural and industrial residues, this technology not only promotes carbon recycling but also reduces pressure on arable land and expands the range of available renewable products. Consolidation of nature-based technologies is crucial for achieving global carbon emission reduction goals and for building a truly sustainable bioeconomy based on technologies that replicate processes established over millions of years. The advancement of -omics tools, such as genomics, transcriptomics, proteomics, and metabolomics, also offers considerable potential to the evolution of fungal coculture systems. These technologies, for example, facilitate the identification of metabolic pathways involved in bioprocesses and the discovery of enzymes as well as metabolites with potential industrial applications, such as in pathogen biocontrol and bioproduct development. Such applications can drive innovation in biotechnology and promote sustainability in industrial processes.

This review explores the potential of fungal coculture as a viable and efficient approach for lignocellulosic biomass degradation and highlights the main challenges and barriers faced in implementing this technology, as well as the potential contributions to a sustainable bioeconomy.

## 2. Importance and Potential of Biomass

The global transportation sector remains highly dependent on fossil fuels, consuming approximately 65% of the world’s refined oil [6]. In this context, biorefineries, which are industries capable of converting lignocellulosic biomass into various products, such as fuels, energy, feed, or biochemicals, are essential to the bioeconomy concept.

Although the definition of the bioeconomy continues to evolve, it encompasses knowledge-driven economic activities that leverage sustainable biological resources to promote industrial development, while also ensuring food security, health, and environmental protection [14,15]. In Brazil, for example, the bioeconomy considers sustainability as well as the valorization of biodiversity [16]. Brazil holds a prominent position on the global bioeconomy stage, given the hotspots of genetic diversity across its distinct biomes, the vast arable land area, a favorable climate for agriculture, and expertise in biofuel production [15,17]. Additionally, in socioecological terms, Brazil benefits from the knowledge of indigenous groups, who have practiced bioeconomic principles long before the Portuguese colonization [18,19]. With regard to biodiversity, Brazil boasts six distinct biomes (Amazon, Caatinga, Cerrado, Atlantic Forest, Pantanal, and Pampa), a coastline spanning 7491 km, and 12 major hydrographic regions. Preservation of such diversity offers considerable potential for the supply of renewable raw materials for a wide range of applications.

**Figure 1:**
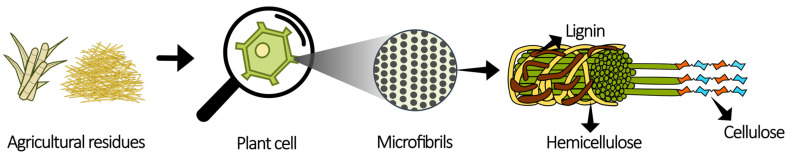
Schematic overview of the structure of agricultural residues, highlighting the intricate composition of cellulose chains within the plant cell wall.

Lignocellulose holds significant potential for a wide range of industrial applications (Figure 1). Historically, it has been used as an energy source, with wood serving for centuries as the most widely used raw material for fire generation. During the Industrial Revolution, as energy demands increased, fossil fuels gradually replaced wood. Since the mid-20th century, however, concerns over pollution and fossil fuel depletion have fueled a growing interest in biomass as a renewable energy source [20]. Today, lignocellulosic residues are utilized to produce second-generation biofuels, bioplastics, nanomaterials, and other value-added products, contributing significantly to the bioeconomy and sustainability initiatives [21]. Biomass conversion is carried out in biorefineries, where lignocellulosic feedstocks undergo a series of processes, including collection, pretreatment, hydrolysis, fermentation, transformation, and product recovery.

Despite the recognized potential of sugarcane bagasse for the production of second-generation (2G) ethanol, its global application remains predominantly confined to electricity generation via combustion [22]. In Brazil, only a small fraction of this biomass is utilized for 2G ethanol production, primarily by the companies Raízen and GranBio. For instance, in 2019, Raízen produced approximately 13,200 tons of 2G ethanol from sugarcane bagasse [23]. The disparity between the technical potential and actual implementation is largely attributed to limitations in biocatalytic technology for enzymatic cocktails, as well as limited investigation of optimization approaches for conversion of bagasse into ethanol and other value-added products [22]. Notably, the technology employed at Raízen for 2G ethanol production was originally developed by the Canadian company Iogen Corporation (Ottawa, ON, Canada) [24]. Given the specific physicochemical characteristics of sugarcane bagasse produced under diverse biotic and abiotic conditions across cultivation sites in Brazil, and in pursuit of national autonomy in scientific, industrial, and biodiversity domains, there is an urgent need to invest in research focused on the biotransformation of lignocellulosic residues within the context of biorefinery models.

The global industrial enzyme market is valued in excess of USD 5.0 billion [25], with recent growth driven by advances in the bioeconomy [26]. Fungi are key producers of enzymes involved in plant cell wall biotransformation; however, a single strain often lacks the full enzymatic repertoire required for efficient biomass saccharification [26,27]. In this context, co-cultivating fungal consortia with complementary genetic and phenotypic traits offers a promising strategy to enhance enzyme diversity and activity [26]. This approach may improve hydrolysis efficiency, increase fermentable sugar yields, and reduce operational costs [28]. Recent studies support the potential of fungal coculture for valorizing sugarcane bagasse. For example, cocultures of *Trichoderma longibrachiatum* and *Thermothelomyces thermophilus* produced efficient enzymatic cocktails for the saccharification of various sugarcane varieties [29], relevant enzymes, and citric acid under controlled conditions [30]. These findings highlight the strategic importance of developing national technologies based on fungal consortia to produce enzymes and high-value-added byproducts from agro-industrial residues such as sugarcane bagasse.

The employment of biomass as a feedstock in biorefineries not only enables the production of biofuels and value-added chemical compounds but also significantly reduces environmental impacts compared to residue disposal and fossil fuel consumption. This approach maximizes the utilization of organic residues (biomass), thereby minimizing pollution, particularly greenhouse gas emissions. Moreover, it ensures a continuous supply of renewable fuels to meet energy demands, supports the generation of chemical compounds for application in the chemical and pharmaceutical industries, and enhances the production and utilization of by-products [31]. Nevertheless, fully harnessing such residues remains challenging, particularly regarding the development of efficient conversion technologies that ensure economic viability while mitigating environmental impacts. Continued research and innovation in this field are crucial for optimizing the use of lignocellulosic biomass and advancing sustainable agricultural and industrial practices.

## 3. CAZYmes and Their Mode of Action in Plant Cell Wall Degradation

### 3.1. Plant Cell Wall Polymer Components

Cellulose is a linear microcrystalline homopolymer with a helical structure and the chemical formula H(C_6_H_10_O_5_)_n_OH, composed entirely of glucose units linked by β-1,4 glycosidic bonds. Its various hydrogen bonding systems influence the packing density, shaping its three-dimensional arrangement through intra- and intermolecular interactions. This structural organization directly affects its properties: hydroxyl groups in amorphous regions are more accessible and reactive, whereas crystalline regions, reinforced by strong intermolecular bonds, are less accessible [32,33]. Hemicellulose is made up of a heterogeneous series of polysaccharides that differ in their structure, branching, type, and distribution of glycosidic bonds. In general, they are classified into four types: (i) xylan, (ii) mannan, (iii) xyloglucan, and (iv) mixed-linkage glucan.

Xylan is the most abundant hemicellulose in the cell walls of terrestrial plants, accounting for over 30% of the total dry weight and approximately 20% of the primary walls. It is a heteropolymer with a backbone of β-(1→4)-D-xylopyranose, which may include 4-O-methyl-α-D-glucopyranosyl acid and α-L-arabinosyl as side chains. Based on these side chains, xylan is classified into three main types: (1) glucuronoxylan, (2) glucuronoarabinoxylan, and (3) arabinoxylan. Sugar modifications such as methylation, acetylation, and feruloylation play a role in protecting polysaccharides from glycosyl hydrolases and regulating cell wall extensibility [34]. The roles of these substitutions are not yet fully understood, but growing evidence suggests that they influence xylan’s interaction with other cell wall components, such as cellulose and lignin, while also contributing to specialized functions within the cell wall. Uniform substitutions can facilitate folding and bonding on the surface of cellulose fibrils, whereas xylan, with more complex substitutions, plays a structural role in the matrix and interacts with other cell wall components [35].

Mannan is classified into four groups based on the sugars present in the β-1,4-linked backbone and the amount of α-1,6-linked galactose residues: (1) homomannan, a linear polymer composed primarily (>90%) of D-mannose; (2) glucomannan, which has a backbone of D-mannose with D-glucose; (3) galactomannan, which contains a D-mannose backbone with more than 5% D-galactose residues; and (4) galactoglucomannan, a glucomannan variant that includes more than 5% D-galactose residues. The backbones of glucomannans and galactoglucomannans are esterified with O-acetyl groups at the C2 and C3 positions of hexoses. The structure and abundance of mannans vary significantly between species, tissues, and developmental stages [36,37].

Xyloglucan consists of a β-1,4-linked D-glucose backbone, frequently substituted at the O-6 position with α-D-xylose residues. These xylose side chains may be further branched at the O-2 position with additional sugar residues, depending on the plant species. The sugars involved in these modifications include β-D-galactose, β-D-galacturonic acid, α-L-arabinofuranose, α-L-arabinopyranose, and β-D-xylose. Additionally, galactose side chains can be further modified at the O-2 position with α-L-fucose. The xyloglucan backbone adopts two main conformations, XXXG and XXGG, where X represents a xylosylated glucose and G a non-xylosylated glucose [38,39]. Finally, mixed-linkage glucans are linear polysaccharides composed of glucose monomers, with segments of three to four glucose units linked by β-1,4 bonds interrupted by β-1,3 bonds. Although structurally similar to cellulose, their solubility is attributed to the presence of β-1,3 linkages, which disrupt the linear arrangement of glucose fibrils [38].

Pectin is characterized by the presence of galacturonic acid (GalA) linked at position 4 of the polymer backbone and consists of four main subclasses: homogalacturonan, a homopolymer of GalA linked by α-D-1,4, which can be methyl-esterified at the C-6 position; rhamnogalacturonan I, which has a GalA backbone alternating with rhamnose (-α-1,4-D-GalA-α-1,2-L-Rha-), with rhamnose potentially replaced by galactose, arabinose, or other sugars; rhamnogalacturonan II, the most complex form, consisting of a homogalacturonan backbone and more than 13 different sugars in various branches; and xylogalacturonan, which has a homogalacturonan backbone with xylose branches. Additionally, pectin can be O-acetylated, with the quantity and position of these acetyl groups varying depending on the plant tissue and developmental stage. An essential functional property of pectin is its ability to form gels through hydrogen bonds, cross-bridging by divalent cations (Ca^2+^), and hydrophobic interactions between the polymer chains [40,41]. The term ‘methyl-esterified’ in pectin refers to methoxylation, which is the esterification of the carboxyl groups of galacturonic acid with methanol.

**Figure 2:**
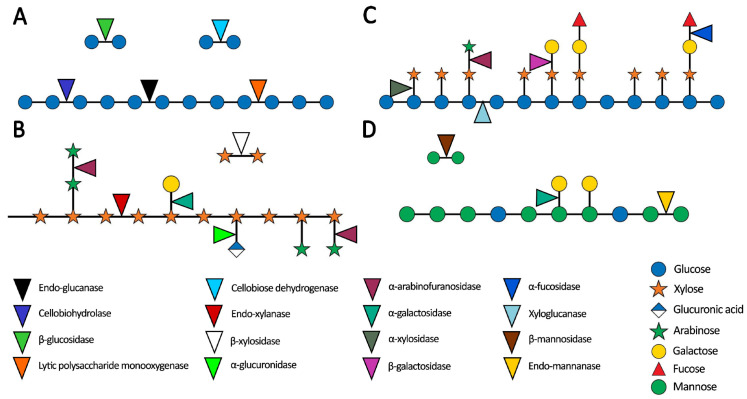
Simplified model illustrating the enzymatic degradation of key cell wall components: (**A**) cellulose, (**B**) heteroxylan, (**C**) xyloglucan, and (**D**) heteromannan. Arrows indicate specific sites of enzymatic action. The structures were generated using the DrawGlycan-SNFG software, version 2.0 [46].

Lignin is a heterogeneous macromolecule composed of phenylpropane derivatives, forming a dense three-dimensional network of phenylpropanoid units: p-hydroxyphenyl (H), guaiacyl (G), and syringyl (S). Each of these units derives from the monolignols p-coumaryl alcohol, coniferyl alcohol, and sinapyl alcohol, respectively. The S units have methoxy substituents at the five positions, which limit certain intermolecular bonding. Lignins possess a variety of C-O and C-C bonds, including β-O-4, β-5, β-β, 5-5, 4-O-5, β-1, and α-O-4. Lignins with a low content of S units tend to form more cross-links, resulting in a denser molecular network that is more difficult to degrade. The composition of lignin varies across plant species: softwoods predominantly contain G lignin, hardwoods are mainly composed of GS lignin, and grasses feature HGS lignin [42]. In native biomass, lignin and carbohydrates are chemically linked, forming lignin–carbohydrate complexes (LCC) that are crucial for the structure of wood. In conifers, all lignin molecules are linked to carbohydrates, whereas, in hardwoods, part of the lignin is mainly with hemicellulose. The extent of carbohydrate-lignin association increases sequentially in grasses, hardwoods, and softwoods [43,44]. These bonds form during lignin biosynthesis, when nucleophiles associate with reactive intermediates arising from the oxidation of p-hydroxycinnamoyl alcohol. The bonds between lignin and carbohydrates serve to protect and reduce the accessibility of cellulose to enzymatic action, thereby decreasing the efficiency of biomass degradation [45]. Carbohydrates involved in these bonds can include cellulose, hemicelluloses, such as xylan, glucuroxylan, arabinoxylan, and glucomannan, and homogalacturonan pectin softwoods [43,44]. Figure 2 illustrates the enzymatic degradation of key cell wall components.

### 3.2. Enzymatic Degradation of the Plant Cell Wall

Microorganisms produce various types of carbohydrate-active enzymes (CAZymes), each specialized in breaking down specific components of the plant cell wall. Among these microorganisms, saprophytic fungi, particularly those from the Ascomycete and Basidiomycete phyla, are exceptionally efficient biological decomposers due to their diverse array of CAZymes, which have evolved to degrade plant cell walls effectively [47,48]. The CAZy database classifies these enzymes based on (i) amino acid sequence similarity; (ii) protein folding; (iii) enzymatic mechanism. Currently, six classifications are described: glycosyl hydrolases (GHs), glycosyl transferases (GTs), polysaccharide lyases (PLs), carbohydrate esterases (CEs), auxiliary activity (AA), and carbohydrate-binding modules (CBMs). Briefly, GHs catalyze the hydrolysis of glycosidic bonds; GTs catalyze the formation of glycosidic bonds; PLs break down polysaccharides containing uronic acid; CEs catalyze the N- or O-acylation of substituted polysaccharides; AAs are composed of enzymes (mainly redox) that break down or build complex carbohydrates; and CBMs are non-catalytic proteins that facilitate substrate binding [47,49]. Table 1 summarizes the role of these enzymes in the specific degradation of plant cell wall components.

The degradation of cellulose by fungi requires four main groups of enzymes: endoglucanases (EGLs), cellobiohydrolases (CBHs), β-glucosidases (BGLs), and auxiliary enzymes (AAs). EGLs act on the amorphous regions of cellulose, generating reducing and non-reducing chain ends. CBHs release cellobiose from these chain ends, with CBH I targeting the reducing ends and CBH II acting on the non-reducing ends. BGLs then hydrolyze cellobiose into glucose. Auxiliary enzymes, such as lytic polysaccharide monooxygenases (LPMOs) and cellobiose dehydrogenases (CDHs), facilitate oxidative cleavage of cellulose, enhancing its breakdown [50,51]. The AA enzymes involved in cellulose degradation and its derivatives play a crucial role not only in directly breaking down cellulose but also in enhancing the activity of other cellulose-degrading enzymes, thereby increasing overall enzymatic efficiency [52]. Xylan degradation involves the coordinated activity of multiple glycosidases and auxiliary enzymes. The primary enzymes acting on the xylan backbone are endo-β-1,4-xylanases and β-xylosidases, which work synergistically with side-chain enzymes, including α-L-arabinofuranosidases, α-1,2-glucuronidases, acetylxylanesterases, β-xylobiohydrolases, and esterases [38,53]. Esterases, which catalyse the hydrolysis of ester bonds between a xylopyranose residue and its acetyl substituent, belong to the carbohydrate esterase (CE) family. Within the CAZy classification, these enzymes are grouped into CE families from 1 to 7 and 12. Feruloyl esterases, classified under the CE1 family, hydrolyze ferulic acid residues esterified to arabinose in xylan side chains. Ferulic acid residues are esterified via an O-ester linkage to the hydroxyl groups of arabinose. Although initially identified by their activity on ferulic acid, they can also release other phenolic acids. Additionally, glucuronyl esterases from the CE15 family hydrolyse the ester bond between glucuronic acid and lignin in glucuronoxylan, while acetyl esterases from the CE16 family function as exodeacetylases, releasing acetic acid from the non-reducing ends of xylopyranosyl residues [54,55].

The complete degradation of mannan requires enzymes with various activities, including β-mannanase, β-mannosidase, α-galactosidase, β-glucosidase, and acetyl mannan esterase. β-Mannanases are endo-hydrolases that break random or specific bonds between D-mannoses in the polymer backbone, creating new ends. β-Mannosidases release D-mannose or D-glucose from the non-reducing ends of the products produced by mannanases and are crucial for fully hydrolyzing mannan. β-Glucosidases release D-glucose units from the non-reducing ends of the oligomers formed by β-mannanases. α-Galactosidases release terminal galactose molecules linked to α-1,6 galactooligosaccharides and other substrates. Acetylmannan esterases specifically remove acetyl groups from mannan oligomers, enabling the backbone-cleaving enzymes to act effectively [56]. Additionally, some LPMOs from the AA9 family can be active on glucomannan [57].

Xyloglucanases are enzymes that hydrolyze the β-1,4-glucan backbone of xyloglucan, a hemicellulosic polysaccharide in the plant cell wall. True endoglucanases are highly specific, but, depending on the degree of polymer substitution and enzyme specificity, the main chain can also be degraded by endo-β-1,4-glucanases, which are commonly involved in cellulose metabolism [58]. Alongside xyloglucanases, a diverse set of enzymes is involved in xyloglucan degradation. α-Xylosidases remove xylopyranosyl side chains attached to glucopyranosyl residues and can also act on isoprimeverose, while isoprimeverose enzymes specifically release isoprimeverose from the non-reducing ends of xyloglucan oligosaccharides. β-Glucosidases hydrolyze the non-reducing glucosyl portion of xyloglucan-derived oligosaccharides. Additionally, α-fucosidases and β-galactosidases are responsible for removing L-fucose and galactose residues, respectively, further contributing to xyloglucan deconstruction [58,59,60]. Additionally, some LPMOs from the AA9 and AA14 families have been shown to act on xyloglucan [61]. The primary enzymes responsible for degrading mixed-linkage glucans are β-1,3-1,4-glucanases, also known as lichenases, which specifically cleave β-1,4 glycosidic bonds at 3-O-substituted glucosyl residues. Additionally, β-1,4-glucanases (endoglucanases) hydrolyze (1→4)-β-D-glucosidic bonds in cellulose, lichenin, and cereal β-D-glucans [62].

Pectin hydrolysis involves the coordinated action of various pectinolytic enzymes, classified into three main types based on their function: protopectinases, esterases, and depolymerases. Protopectinases catalyze the solubilization of protopectin, as they degrade the insoluble substrate, producing a highly polymerized soluble pectin; esterases separate the methoxyl and acetyl residues from pectin to produce pectic acid and can be classified as methylesterases and acetylesterases; and depolymerases, which degrade pectin by breaking α-1,4 glycosidic bonds in D-galacturonic acid. This group includes polygalacturonases, which hydrolyze pectin in the presence of water, and pectin lyases, which cleave pectin through a trans-elimination reaction, generating unsaturated oligo-galacturonides [51,63].

Lignin bioconversion occurs through a consortium of enzymes, each playing a distinct role in its degradation. These include AA1 family enzymes (laccases), AA2 family enzymes (lignin peroxidases, manganese peroxidases, versatile peroxidases), O-demethylase, the β-etherase system, CE15 family glucuronoyl esterases, and indirectly acting enzymes such as aryl alcohol oxidases and glyoxal oxidase. Laccases are copper-containing enzymes that oxidize phenolic lignin compounds, using molecular oxygen as an electron acceptor and producing water. The AA2 family peroxidases play a central role in lignin degradation, utilizing H_2_O_2_ as a co-substrate to catalyze the oxidation of high-redox-potential aromatic compounds. Within this family, lignin peroxidases (LiPs) perform one-electron oxidation of phenols, aromatic amines, ethers, and polycyclic aromatics, generating cationic radicals. Manganese peroxidases (MnPs) oxidize Mn^2+^ to Mn^3+^, which acts as a diffusible mediator targeting lignin’s phenolic structures. Versatile peroxidases (VPs) combine the properties of LiPs and MnPs, directly oxidizing lignin or using mediators such as Mn^2+^ and veratryl alcohol, making them highly adaptable to different environments [42,64]. Other enzymatic systems also contribute to lignin degradation. O-demethylases remove methoxy (-OCH_3_) groups from lignin’s aromatic rings, increasing the number of phenolic hydroxyl groups. This reaction releases methanol as a by-product and enhances lignin’s solubility and reactivity. Enzymes involved in this process include 3-, 4-, and 5-O-demethylases, methanol oxidase, and vanillate hydroxylase, with laccases and peroxidases assisting indirectly [65].

The bacterial β-etherase system cleaves β-O-4 bonds in lignin through a three-step enzymatic process: short-chain dehydrogenases oxidize the Cα position in lignin dimers, β-etherases (glutathione-S-transferases, GSTs) break β-O-4 bonds using glutathione (GSH) as a cofactor, and β-glutathione thioetherase removes glutathione from the resulting intermediates. Glucuronoyl esterases hydrolyze the ester bond between glucuroxylan and lignin, facilitating the breakdown of lignin–carbohydrate complexes [54]. Additionally, aryl alcohol oxidases and glyoxal oxidases generate H_2_O_2_, which is essential for ligninolytic peroxidases. Aryl alcohol oxidases act on aromatic alcohols and aldehydes derived from lignin or fungal metabolism, while glyoxal oxidases target aliphatic aldehydes, α-hydroxycarbonyl, and α-dicarbonyl compounds, likely formed through C3 side chain oxidation in lignin [66,67]. Recent studies suggest that other oxidative enzymes may also contribute to lignin degradation. Dye-decolorizing peroxidases have demonstrated oxidative activity on lignin-derived compounds, though without altering carbohydrate profiles or affecting β-ether lignin dimers. Furthermore, fungal short polyphenol oxidases may act on lignin-derived phenols, though their ability to degrade lignin itself remains unconfirmed [68,69,70].

## 4. Limitations in Fungal Monoculture-Based Lignocellulose Biodegradation Approaches

The decomposition of organic matter began with the emergence of the earliest life forms on Earth, from approximately 3.5 to 3.8 billion years ago. As life evolved into more complex multicellular organisms, including plants and animals, the quantity and diversity of organic matter available for decomposition increased substantially. Decomposition reflects billions of years of coevolution among diverse organisms and represents a critical ecological process fundamental for the establishment and maintenance of life as we know it. The evolution of higher plants was enabled by the presence of lignin, a complex phenolic polymer that provides structural rigidity, hydrophobicity, and resistance to degradation. This recalcitrant nature necessitates a sophisticated enzymatic arsenal for its breakdown, as well as for the subsequent degradation of the protected fractions of cellulose and hemicellulose. The efficient biodeconstruction of the plant cell wall began approximately 400 million years ago and involves a complex and diverse community of saprophytic organisms, including fungi, bacteria, protozoa, invertebrates, autotrophic algae (including lichens), bryophytes, and vascular plants [71,72].

Among the organisms involved in the biodeconstruction of lignocellulosic biomass, fungi have developed a highly efficient bioconversion mechanism that involves the ability to secrete extracellular enzymes and actively transfer carbon, nutrients, water, and oxygen through highly branched hyphal networks. Within the kingdom Fungi, the phyla *Ascomycota* and *Basidiomycota*, belonging to the sub-kingdom *Dikarya*, are the most abundant in surface soils and are highly specialized in the biodeconstruction of lignocellulosic biomass. While Ascomycetes are more efficient at decomposing cellulose and hemicellulose, Basidiomycetes, especially the white rot fungi, are efficient in lignin degradation [73]. Ascomycetes generally rely on a battery of hydrolytic enzymes, such as endoglucanases (GH5, GH12), exoglucanases (GH6, GH7), β-glucosidases (GH1, GH3), and hemicellulases including xylanases (GH10, GH11), arabinofuranosidases (GH51, GH54), and mannanases (GH26). However, they typically lack high-efficiency oxidative enzymes for lignin depolymerization, such as lignin peroxidases (LiPs, manganese peroxidases (MnPs), and versatile peroxidases (VPs), limiting their capacity to initiate the breakdown of intact lignocellulosic matrices [74,75].

Despite the great potential of fungi in lignocellulosic biomass degradation, fungal biodiversity remains largely unexplored. Current estimates suggest that there are between 2.2 and 3.8 million fungal species. However, only 164,427 fungal species have been formally described to date (https://www.speciesfungorum.org, accessed 7 April 2025), of which approximately 93,000 are ascomycetes [76] and 40,000 are basidiomycetes [77]. According to [77], the total number of basidiomycete species worldwide is estimated to range from 1.4 to 4.2 million. Fungal species involved in plant biomass decomposition utilize a variety of strategies to degrade lignocellulose. Based on these strategies, they have traditionally been classified into three functional groups: white rot, brown rot, and soft rot fungi. White rot fungi (WRF) are distinguished by their ability to degrade lignin through the employment of oxidative enzymes such as peroxidases. The resultant exposure of underlying carbohydrates then enables access of CAZYs such as glycoside hydrolases (GHs), which act on hemicellulose and cellulose. In contrast, brown rot fungi (BRF) depolymerize the cell wall through the employment of reactive oxygen species, selectively breaking down components and releasing soluble sugars. While both white rot and brown rot fungi belong to the class *Agaricomycetes* within the phylum *Basidiomycota* [78], soft rot fungi (SRF) are classified within the phylum *Ascomycota* and employ alternative and less well-characterized mechanisms, such as cavitation or erosion, to access sugars in the lignocellulosic matrix [78,79]. This traditional functional classification of rot fungi is now regarded as somewhat limited [78], with the adaptive flexibility observed in certain species, particularly among the white rot fungi, challenging the dichotomy between white and brown rot, leading to the concept of grey rot [80]. This term underscores the complexity of fungal degradation processes, where substrate selectivity and genes involved do not follow a rigid pattern, highlighting the multifaceted nature of fungal decomposition mechanisms and their broader implications for the global carbon cycle.

The phylum *Ascomycota* is divided into three subphyla: *Saccharomycotina* (budding yeasts), *Pezizomycotina* (filamentous fungi), and *Taphrinomycotina* (fission yeasts). Among these, *Pezizomycotina* exhibit the greatest taxonomic and ecological diversity, encompassing numerous economically and ecologically important species, including pathogens, decomposers, and symbionts [81]. A well-studied genus among the Ascomycota is *Aspergillus*, whose species are widely utilized in enzyme production for the degradation of plant polysaccharides. Members of this genus exhibit a generalist lifestyle with regard to carbon utilization. This metabolic diversity enables *Aspergillus* species to colonize a wide variety of habitats, reflecting specific adaptations to distinct ecological niches [82]. Another notable genus within the *Ascomycota* is *Trichoderma*, which has been extensively studied regarding its cellulolytic capacity. *Trichoderma reesei*, for instance, is widely used in industrial enzyme production and expresses a well-characterized enzymatic cocktail of endo- and exoglucanases, xylanases, and β-glucosidases [83]. However, its limited ligninolytic activity constrains its ability to degrade intact lignified biomass, requiring either prior delignification or synergistic interactions with other lignin-degrading organisms.

Basidiomycete fungi also play a fundamental role in the global carbon cycle. Saprobic basidiomycetes, particularly those in the order *Agaricales*, are primary agents of plant biomass degradation in soils and forest ecosystems. These fungi are traditionally classified based on their effects on and preferential degradation of lignocellulosic polymers. WRF depolymerize and mineralize lignin, thereby enabling access to cellulose and hemicellulose. BRF primarily target cellulose, degrading wood polysaccharides while partially modifying lignin. SRF predominantly degrade polysaccharides, with only limited ligninolytic capacity. WRF possess genes encoding class II ligninolytic peroxidases, while BRF, which have evolved from white rot ancestors, are considered to have lost ligninolytic genes [84]. Over the course of approximately 170 million years, the *Agaricales* have undergone extensive diversification, enabling them to colonize and degrade a range of lignocellulosic substrates, including grass and forest litter. This adaptive evolution on different substrates has led to the emergence of diverse saprotrophic and biotrophic lifestyles [84]. The Fenton reaction, a key mechanism employed by BRFs for wood degradation, generates reactive oxygen species (ROS), notably hydroxyl radicals (•OH), via the reaction between ferrous iron (Fe^2+^) ions and hydrogen peroxide (H_2_O_2_) under acidic conditions. Fungal metabolites such as oxalate and siderophores chelate ferric iron (Fe^3+^) and reduce it to Fe^2+^, sustaining the continuous production of ROS. Fungi can enzymatically supply H_2_O_2_ via AA3 GMC oxidases, AA5 oxidases, or possibly through non-enzymatic processes. Phenolic compounds and quinones, either derived from plant lignin or produced by fungi, assist in electron transfer and Fe recycling, with laccases participating in these redox reactions. The initial oxidative disruption of the lignocellulose matrix enhances enzymatic accessibility while also protecting polysaccharide-degrading enzymes from ROS-mediated damage [85]. Among basidiomycetes, fungi with similar ecological lifestyles tend to possess convergent lignocellulose-degrading enzymatic machineries, regardless of their phylogenetic lineage. WRFs typically possess a higher number of enzymes, such as LPMOs and CBM1, in the AA9 family. Conversely, BRF tend to possess fewer LPMO genes (ranging from 8 to 30). The abundance of LPMO genes correlates with the WRF lifestyle of wood and litter decomposers [84]. Another notable difference related to lifestyle is the presence of extracellular enzymes activated by ROS, such as LPMOs. WRFs also encode numerous genes for AA2 peroxidases (class II) and specific members of the AA3-GMC enzyme family responsible for H_2_O_2_ production [85].

The plant cell wall is a highly modular and heterogeneous structure, composed of various proportions of polysaccharides, associations, and covalent linkages with lignin, as well as diverse intra- and intermolecular interactions among its components. This structural complexity, combined with the intricate mechanisms of lignocellulose biodegradation exhibited by Ascomycetes and Basidiomycetes, reflects a long-standing coevolutionary process that remains largely unexplored and poorly understood. In this context, fungal coculture systems represent a promising strategy to overcome the inherent limitations of fungal monocultures. By combining ligninolytic Basidiomycetes, capable of oxidative lignin depolymerization, together with hydrolytic Ascomycetes enriched in glycoside hydrolases, it is possible to establish a sequential or synergistic degradation of the plant cell wall [85]. Consequently, the rational design of fungal consortia holds considerable potential for biotechnological applications in the fields of bioenergy production, bioremediation, and within broader circular economy frameworks.

## 5. Fungal Coculture

Microorganisms coexist in nature in dynamic environments, with complex interactions influencing essential ecological processes. Symbiotic relationships among fungi, which include competition, mutualism, commensalism, and antagonism, are key to maintaining a balance in the fungal community and the organic matter decomposition cycle [86]. Such interactions can also be replicated under controlled laboratory conditions. Here, fungal coculture can be defined as the simultaneous cultivation of different fungal species or strains in a single culture medium, with growth maintained under controlled and axenic conditions. This approach enables investigation of the dynamics between examined fungal combinations as well as exploration of their biotechnological potential in industrial applications [87]. These interactions may result in competition for resources, such as nutrients and space, or a symbiosis that promotes mutual growth and utilization of the growth media.

Fungal interactions can be antagonistic, as demonstrated in the case of the basidiomycete species *Trametes versicolor* and *Ganoderma applanatum* [88]. In such interactions, one organism may secrete bioactive compounds that inhibit the growth of the other, leading to the development of a distinct confrontation zone. Similarly, actinomycetes can exhibit antagonistic behavior towards cocultured fungi by releasing metabolites that suppress growth [89]. Such interactions, which are involved in competitive niche exclusion, involve secreted fungal secondary metabolites. These low molecular weight molecules, which are classified into polyketide, non-ribosomal, alkaloid, and terpene chemical categories, in addition to influencing ecological dynamics, also offer potential as antimicrobial compounds for direct control of plant pathogens via biological control or via activation of plant host defenses following induced systemic resistance. Fungal secondary metabolites also include organic acids and biocatalysts such as enzymes [90]. Their biosynthesis is often tightly regulated and triggered by environmental cues, including the presence of competing organisms. In this context, coculture strategies have been increasingly employed to activate the production of metabolites that are typically not expressed under synthetic media or monoculture conditions, but which may exhibit unique bioactive properties under specific environmental stimuli.

In addition to antagonistic interactions, mutualism can also occur in fungal cocultures. One example is the interaction between *Grifola umbellata* and *Armillariella mellea*, wherein the formation of *G. umbellata* sclerotia and continued fungal growth depend on nutrients provided by *A. mellea* [91]. From a bioeconomic perspective, mutualistic interactions are critical for unlocking enzymatic profiles that are otherwise repressed in monoculture systems. Such interactions can lead to functional specialization, whereby each fungus contributes distinct enzymatic activities in a coordinated manner, thereby optimizing the degradation of complex substrates. This dynamic not only enhances process efficiency but also enables the development of tailored enzyme consortia for specific industrial applications.

Such applications have the potential to reduce production costs, thereby increasing the economic viability of industrial processes. Enzymatic analyses of fungal coculture have demonstrated significant potential for enhancing lignocellulose degradation, particularly through the involvement of the efficient biodegrader *Trichoderma reesei* [92,93]. This Ascomycete species is frequently cocultured with other fungi to compensate for its low secretion of β-glucosidases—for instance, with *Aspergillus niger*, which improves overall saccharification performance [94,95,96]. Recent studies have further confirmed that coculture strategies, such as those involving *T. reesei* and *Aspergillus brasiliensis*, can significantly increase enzymatic activities, including CMCase and pectinase [95], while also enhancing sugar yields from lignocellulosic substrates such as wheat bran [96]. Key fungal consortia applied to lignocellulosic biomass are summarized in Table 2.

**Figure 3:**
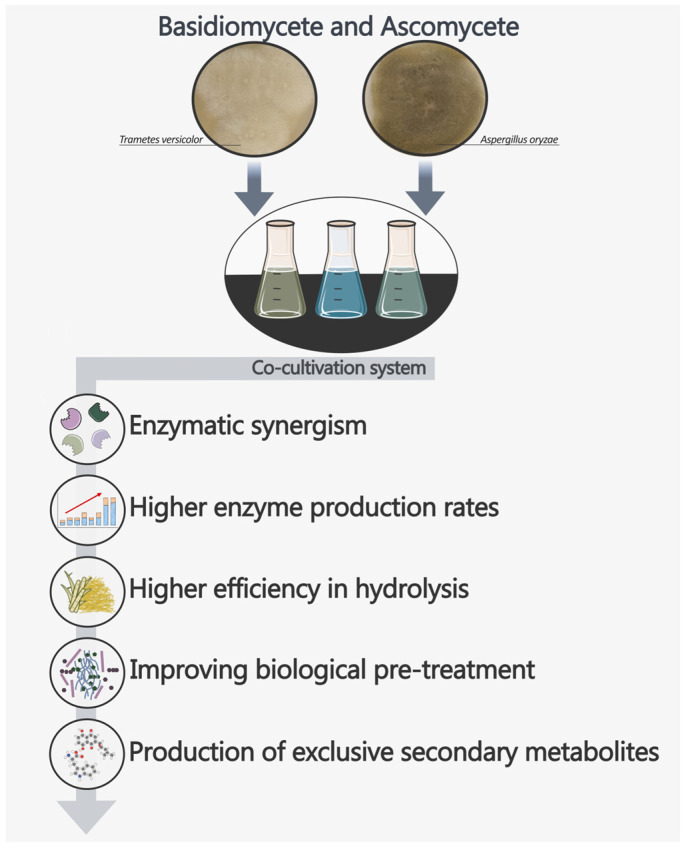
Summary of biorefinery applications of Ascomycete and Basidiomycete fungal coculture systems.

In addition to more common ascomycete–ascomycete combinations, cocultures involving basidiomycetes have gained attention due to their superior lignin-degrading capabilities. Basidiomycete species such as *Coprinus comatus*, *Phanerochaete chrysosporium*, and *Pleurotus eryngii* have demonstrated enhanced enzymatic activities for both saccharification and lignin breakdown, making them valuable contributors to bioconversion processes [97,98,99,100]. Lignocellulose biodegradation mediated by Ascomycete and Basidiomycete fungi in coculture systems offers significant biotechnological potential for application in sustainable industrial processes, effectively mimicking natural biological processes (Figure 3). While Ascomycetes effectively secrete hydrolytic enzymes for holocellulose degradation, lignin breakdown is more efficiently carried out by white-rot Basidiomycetes [101].

Fungal coculture also stands out as an environmentally and economically viable alternative for biomass conversion when compared to traditional monoculture and conventional pretreatment processes. Conventional physicochemical pretreatment processes, particularly alkaline methods, are energy-intensive and often lead to the formation of toxic compounds such as furfural, which can inhibit enzymatic activity and impair downstream hydrolysis. A notable example is the cocultivation of *Aspergillus nidulans* by [102], which, despite yielding lower hemicellulose conversion than alkaline pretreatment, avoided the production of inhibitory residues such as sodium hydroxide, which hampers cellulose hydrolysis. Coculture also improves resource efficiency by enabling the simultaneous production of diverse enzymes and facilitating biomass hydrolysis and fermentation within a single reactor. This can significantly reduce operational costs associated with commercial enzymes and the use of aggressive chemical agents. Moreover, the application of agro-industrial residues as substrates in fungal coculture supports circular economy principles, lowers carbon emissions, and mitigates environmental impacts associated with the improper disposal of biomass waste [103]. The use of such residues in biotechnological processes can reduce production costs by 30–40%, while contributing to the sustainable management of approximately 1.3 billion tons of agri-food waste generated annually. Fungal coculture has also demonstrated strong potential in environmental applications. For instance, the association between *Trametes versicolor* and *Phanerochaete chrysosporium* led to an 8.2-fold increase in laccase activity compared to monoculture, improving textile dye degradation through a low-cost, eco-friendly alternative to conventional physicochemical treatments [104]. Overall, fungal coculture of agro-industrial residues enhances the sustainability of biomass conversion processes by reducing carbon footprint, lowering production costs, and promoting full utilization of renewable materials. This approach supports the development of integrated biotechnological platforms aligned with circular economy practices for waste valorization [105].

## 6. Transcriptomic Analysis in Mono- and Coculture Systems

Transcriptomics is the study of the structure, function, and evolution of the transcriptome of an organism under specific conditions [106]. RNA sequencing (RNA-Seq) is a widely utilized method for transcriptome analysis, offering high-throughput and high-resolution quantitative assessment of gene expression and isoform abundance. Complementary approaches also include reverse transcription quantitative PCR (RT-qPCR) for sensitive validation of specific transcripts, and, to a lesser extent today, microarray analysis for transcript profiling in organisms with annotated reference genomes. When applied to coculture systems, transcriptomics serves as a powerful tool for elucidating the molecular basis of interspecies interactions. Coculture, as an important strategy for simulating the natural living conditions of macrofungi, can activate repressed genes or biosynthetic gene clusters through interspecific signaling [91]. By capturing transcriptional responses triggered during such interactions, transcriptomic analyses facilitate the identification of differentially expressed genes involved in the production of secondary metabolites and extracellular enzymes. This not only advances the discovery of novel bioactive compounds and industrially relevant enzymes but also enhances our understanding of fungal communication, metabolic regulation, and ecological adaptation within complex microbial environments.

### 6.1. Transcriptome Analysis of Fungal Monoculture

With regard to fungal monocultures, particularly in lignocellulolytic fungi, RNA-Seq has been extensively employed for the identification of genes involved in primary and secondary metabolic pathways associated with environmental adaptation and enzyme production [107,108,109]. This area of research has significant relevance for biotechnological applications, with implications for the production of industrial enzymes, pharmaceuticals, biopesticides, and for use in waste treatment [110,111]. With concerning to fungal CAZymes involved in biomass degradation, numerous genomics and transcriptomic studies have investigated CAZyme-encoding and regulatory genes in a wide range of ligninocellulolytic fungi, including *Trichoderma reesei*, *Aspergillus niger*, and *Pleurotus ostreatus*. Gene expression profiling in response to specific polysaccharide carbon sources has elucidated enzyme systems specialized for the efficient deconstruction of lignocellulosic substrates [112,113,114,115]. Moreover, RNA-Seq continues to facilitate the discovery of novel CAZymes with unique functions and substrate specificities, offering further potential to enhance the efficiency of biomass deconstruction [116,117].

Transcriptomics is also a valuable tool for the characterization of transcription factors (TFs), proteins that regulate gene expression by modulating the activity of signal transduction pathways in response to environmental or cellular stimuli [118,119]. Although TF repertoires remain largely uncharacterized in many fungal genomes [120], numerous TFs have now been identified across diverse fungal species. Many of these play pivotal roles in regulating genes encoding enzymes involved in plant biomass degradation [121]. In the genus *Aspergillus*, key TFs include XlnR, a principal regulator of (hemi)cellulose degradation; AraR, involved in L-arabinose metabolism; ClrA and ClrB, both associated with cellulose breakdown; and AmyR, which regulates starch degradation. Additional TFs include MalR (maltose metabolism), ClbR (cellobiose utilization), RhaR (pectin degradation and L-rhamnose metabolism), GaaR (pectin degradation and galacturonic acid utilization), InuR (inulin metabolism), GalX and GalR (D-galactose utilization), and CreA, a central component in carbon catabolite repression, which negatively regulates cellulases, hemicellulases, and pectinases [122]. In *Penicillium* spp., TFs regulating cellulase gene expression include ClrB, ClrC, PacC, AreA, and AraR [121]. In *T. reesei*, the regulation of cellulase-related genes is controlled by TFs, such as Xyr1 and Clr1, which are responsive to different carbon sources [123]. Xyr1 (Xylanase regulator 1) is the primary activator of cellulase and hemicellulase gene expression, functioning in part through recruitment of the SWI/SNF chromatin-remodeling complex [124]. Rce1 acts as a repressor by antagonizing the binding of Xyr1 to cellulase gene promoters [125], while RXE1 modulates Xyr1 and consequently affects cellulase gene expression [126]. Moreover, the MAT1-2-1 protein interacts with Xyr1 to influence cellulase gene expression in response to light [127]. In Basidiomycetes, the TF Roc1 has been identified as a key regulator of cellulose degradation in the wood-decaying fungus *Schizophyllum commune*. Roc1 directly regulates genes involved in lignocellulose degradation, including those encoding lytic polysaccharide monooxygenases (LPMOs) [128]. These findings underscore both the conservation and divergence of transcriptional regulation mechanisms across fungal species, with significant implications for understanding and optimizing lignocellulosic biomass degradation.

### 6.2. Transcriptome Analysis of Fungal Coculture

Transcriptomics also represents a powerful approach for investigating fungal coculture systems, enabling the quantification of gene expression changes and enhancing our understanding of interspecies interactions at the molecular level [129]. Fungal interactions, which can be either cooperative or competitive, can significantly alter gene expression, particularly those involved in the biosynthesis of enzymes, secondary metabolites, and other bioactive compounds. Notably, many of these compounds are not expressed under monoculture conditions, highlighting the relevance of coculture in biotechnology and bioproduct development, including the development of biopesticides, pharmaceutical agents, and biofuels.

Several transcriptomic studies have demonstrated the potential of fungal cocultures in enhancing the production of industrially relevant enzymes. Ref. [130], for example, analyzed the transcriptomes of *Penicillium* and *Trichoderma* in coculture, identifying differentially expressed genes encoding lignocellulolytic enzymes, as well as genes involved in secondary metabolite synthesis, relevant for the production of antibiotics. Similarly, ref. [123] observed considerable modulation in expression of genes encoding cellulases, hemicellulases, and xylanases, again in *Penicillium* and *Trichoderma* cocultures, indicating an enhanced capacity for lignocellulose degradation. In contrast, ref. [131] reported only modest increases in CAZyme transcript levels in cocultures involving *Trichoderma reesei*, *Penicillium chrysogenum*, and *Aspergillus niger*, with upregulation mostly in secondary metabolism-associated genes indicative of competitive rather than cooperative interactions. These findings highlight the importance of selecting compatible microorganisms to promote synergistic enzyme production. Despite growing interest, transcriptomic investigations specifically focused on lignocellulose degradation in fungal cocultures remain scarce, with further research warranted to uncover the full enzymatic potential and regulatory mechanisms governing the complex interactions under these conditions.

Beyond enzyme production, transcriptomic analyses of fungal coculture have also been applied to discover antimicrobials and other mechanisms involved in fungal growth inhibition. For instance, coculture of *Aspergillus fumigatus* with *Candida albicans* led to the activation of defense-related genes, including those related to secondary metabolism and antimicrobial compound biosynthesis [132]. Additionally, RNA-seq of the biocontrol yeast *Hyphopichia pseudoburtonii* cocultured with the plant pathogen *Botrytis cinerea* revealed differential expression patterns indicative of antifungal interactions. These included genes involved in histone and ribosome biogenesis, filamentous growth, and nutrient competition, specifically iron, copper, and sugar transport, as well as siderophore activity, resulting in the suppression of *B. cinerea* growth [133].

With regard to TFs in coculture, these regulatory proteins not only play a role in symbiotic and competitive interactions but are also crucial for fungal adaptation to different environments, optimizing survival and substrate utilization. In coculture, the presence of one fungus can induce expression of specific TFs in another, with a resultant modulation of expression of genes involved in the production of enzymes, metabolites, and other defense compounds. RNA-Seq, in addition to facilitating the identification of genes and metabolic pathways uniquely activated or intensified during coculture, has also been useful in mapping TFs involved in the coculture interaction in fungi. Examples, however, of TFs specifically characterized in transcriptomic studies of fungal cocultures remain scarce, particularly those associated with lignocellulose degradation, indicating an important gap in current research.

#### Challenges in Transcriptomic Analysis of Fungal Coculture

While the potential of fungal coculture is considerable, such systems can present challenges, not only with regard to optimization of growth conditions for induction of gene expression and product recovery, for example [88], but also with regard to investigating interactions at the genetic and molecular level. Fungal coculture, for example, can require accurate monitoring and quantification of organisms present in any sample. While DNA amplification methods via PCR or qPCR are often employed to confirm the presence and relative proportions of each fungus in a sample [134], problems may occur if organisms are genetically similar or if primers are not sufficiently specific. Flow cytometry, however, can be applied as an alternative, enabling identification based on morphological and fluorescence characteristics [135].

In transcriptome analysis of coculture, as RNA-Seq reads will typically originate from both organisms, precise mapping of data to high-quality reference genomes for each organism is essential for identifying respective transcripts from each organism. Incomplete genomes, in contrast, can lead to erroneous interpretations following transcript mapping and quantification. In many cases, fungal reference genomes may also be insufficient to ensure accurate analysis, especially for fungi with large and complex genomes [134]. The presence of homologous transcripts or shared DNA sequences also complicates the precise assignment of genes to their respective organisms. To address this, read-mapping tools such as Bowtie or TopHat can be adjusted to differentiate sequences from each organism [108]. Tools such as Cufflinks or Salmon can also be used to assign transcripts to a specific genome based on a predefined reference set. Additionally, information on specific genome characteristics, unique sequences, or isoforms is also crucial for differentiation.

Normalization of RNA-Seq data is also crucial in transcriptomic analysis, particularly in studies involving fungi in coculture. Normalization ensures that observed differences in gene expression are attributed to real biological variations, rather than technical variations. In cocultures, where multiple organisms are present, normalization becomes a challenge because differentiating the contributions of each organism to the transcriptomic profile requires advanced normalization techniques to handle the complexity of mixed samples. For example, one fungus may have a higher number of transcripts, which could mask the gene expression of the other. In this context, normalization needs to account not only for the total RNA quantity but also for the relative abundance of transcripts from each organism. Different normalization methods have been developed to address these challenges. One of the most commonly employed is Total Count Normalization, which adjusts data based on the total number of sequence reads in each sample, assuming that the total number of transcripts is similar across comparable samples. However, this method can be inadequate when the relative abundances of the organisms vary substantially, as is the case in certain cocultures, as it may lead to underestimation or overestimation of the expression of certain genes [136]. A more advanced approach is normalization based on Transcript Per Million (TPM) or Reads Per Kilobase per Million Reads (RPKM), which adjusts the data by considering both gene length and sequencing depth. These methods are useful when working with different organisms in a single RNA-Seq experiment, as they allow for comparison between the expression levels of genes of varying sizes and abundances [137]. Tools such as dual RNA-Seq have also enabled simultaneous analysis of the transcriptome of two organisms, with normalization, for example, that can distinguish gene expression in a host and a fungal pathogen [138]. Additionally, tools such as DESeq2 and edgeR, which are widely used for RNA-Seq data analysis, can be configured to perform normalization that accounts for the specificities of cocultures, adjusting read counts to reflect differences in the abundance of each fungus [139]. When performing RNA-Seq data normalization in fungal cocultures, the availability of high-quality reference genomes for the organisms under study is also essential. Incomplete or poorly annotated genomes can hinder normalization and data analysis, with RNA-Seq reads not correctly mapping to the corresponding genes, leading to inaccurate analysis of gene expression and compromising data interpretation [134]. Ideally, complete and well-annotated reference genomes for the fungi present in the coculture should be utilized, or combined genomic datasets from different sources.

Clearly, while transcriptomic analysis is already a powerful approach for exploring fungal interactions in coculture, the high-throughput technologies employed require substantial resources, including specialized equipment and computational infrastructure. Moreover, expertise in bioinformatics is crucial for data analysis and interpretation. Continued efforts are also required in enhancing bioinformatics tools to facilitate comprehensive analyses of complex fungal interactions.

## 7. Proteomic Analysis of Mono- and Cocultures

The secretome, the subset of the proteome consisting of proteins released by a cell, tissue, or organism, alters its composition in response to environmental conditions. Fungal secretomes offer potential as highly efficient natural biofactories, with secreted enzymes characterized by high specificity and catalytic efficiency, making them strategic for diverse industrial applications given their favorable cost-benefit ratio [140]. Proteomic analyses of fungal secreted proteins provide critical insights into enzyme diversity, metabolic regulation, and interspecies interactions. With the increasing availability of fungal genomes, coupled with advancements in protein separation and identification technologies, significant advances in fungal secretome research have occurred in recent years [141,142,143,144].

Nowadays, fungal secretomic analysis employs high-performance approaches based on mass spectrometry (MS), which enables the precise identification of up to thousands of proteins present in the samples. Two main MS strategies are employed in proteomics: Top-Down, which examines intact proteins, enabling inferences about molecular mass, isoforms, and post-translational modifications (PTMs); and Bottom-Up, which analyzes peptides following proteolytic digestion. The Bottom-Up approach is presently the most widely used, allowing efficient analysis of complex samples and providing comprehensive information about the set of secreted proteins [145,146]. Furthermore, bioinformatics tools are essential for predicting and analyzing protein profiles based on reference genomes. Several technical challenges, however, limit the comprehensiveness of proteomic datasets. These include the vast dynamic range of protein abundances within cells, which hampers the detection of low-abundance proteins, and the inherent difficulties associated with the solubilization, extraction, and analysis of hydrophobic proteins, particularly membrane-associated proteins. Nonetheless, significant advancements in liquid chromatography coupled with tandem mass spectrometry (LC-MS/MS) have considerably enhanced the sensitivity, resolution, and coverage of proteomic analyses. These methodological improvements now permit the detection and quantification of low-abundance, highly glycosylated, and hydrophobic proteins, including integral membrane proteins, thus expanding the proteomic landscape [147].

Studies on protein–protein interactions (PPIs) have explored how proteins modulate signaling pathways and cellular responses within the secretome [148,149]. The integration of proteomic and transcriptomic data enables the mapping of different cellular regulatory levels that affect secretome composition and functionality, enhancing the understanding of metabolic strategies adopted by fungi in response to environmental changes. These networks elucidate interaction dynamics, such as symbiosis or antagonism, demonstrating how PPIs adjust their functions to cooperate or compete for resources. Understanding these relationships in coculture is essential to decipher the molecular pathways underlying adaptive responses and to identify regulatory targets governing specific interactions.

In addition to the analysis of monocultures, proteomic analysis can also be applied to microbial consortia, providing a detailed perspective of the dynamic genotypic and phenotypic features of community members. Analyzing the secretome in coculture, cross-referenced with the reference genomes of the involved organisms, allows identification of the specific contribution of each fungal species, determining the number and profile of proteins associated with each organism. This approach provides detailed insights into the dynamic genotypic and phenotypic characteristics of species within the community. Traditional methods for enzymatic characterization of lignocellulose have resulted in limited knowledge about lignocellulolytic enzymes, and inter- and intra-species interactions during lignocellulosic biomass degradation remain poorly understood. In this context, coculture of fungal strains with high lignin and cellulose degradation capacities emerges as a promising strategy to enhance lignocellulose deconstruction efficiency, discover new enzymes, and elucidate synergistic and cooperative mechanisms. This enables the investigation of protein expression by Basidiomycetes and Ascomycetes on complex lignocellulosic substrates [95].

A recent study by [124] investigated the coculture of *Aspergillus brasiliensis* and *Trichoderma reesei* RUT-C30 on sugarcane bagasse, demonstrating that the sequence of fungal inoculation influences the enzymatic and proteomic profiles of the secretomes. Specific coculture conditions led to enhanced activities of CMCase, pectinase, β-glucosidase, and β-xylosidase, when compared to monocultures. Proteomic analyses identified 167 proteins, and their distribution between species varied depending on the inoculation strategy, highlighting how experimental design can modulate enzyme production in coculture systems.

Other proteomic studies involving fungal coculture for lignocellulose degradation remain limited. Ref. [150], for example, analyzed cocultures of recombinant *Trichoderma reesei* and *Aspergillus niger*, observing increased cellulase activity and more effective degradation of lignocellulosic material compared to individual cultures. Despite these examples, proteomic research specifically addressing fungal coculture on lignocellulosic substrates remains scarce in the literature. This gap highlights the need for further investigation into fungal interactions and enzyme expression under coculture conditions.

## 8. Integration of Multi-Omics Data

Integration of multi-omics data, including transcriptomics, proteomics, and metabolomics, offers a comprehensive view of microbial interactions in co-cultivation systems, facilitating the refinement of CAZyme gene annotations in fungi, advancing understanding of gene regulation, and unraveling the complex interactions within the enzymatic machinery, thereby fostering a deeper understanding of adaptive functions [151,152]. Transcriptomics provides insights into gene expression patterns under co-culture conditions, while proteomics reveals post-transcriptional regulation and the functional protein output. Metabolomics adds another dimension by capturing the biochemical outputs of microbial metabolism, reflecting both intrinsic and interaction-driven processes. Integrating such datasets is complex, as mRNA and protein levels often exhibit only moderate correlation. This discrepancy between mRNA abundance and protein expression is influenced by several factors, including post-transcriptional and post-translational modifications, the differential stability of mRNA, and variations in translation efficiency [134]. Specifically, the physical properties of transcripts, such as the presence of a Kozak sequence, mRNA secondary structure, variability in expression during the cell cycle, and ribosome density, can all impact translation. Codon bias, often quantified by the codon adaptation index (CAI), also affects translational efficiency by modulating ribosome decoding speed and tRNA availability [153,154]. Additionally, post-translational modifications, such as phosphorylation and ubiquitination, influence protein stability. To address the challenges of integrating datasets, careful data alignment is essential, requiring strategies such as normalization, time-resolved sampling, and advanced computational tools to handle cross-platform variability. Systems biology approaches, including correlation analysis between gene expression and protein levels [155], protein interaction networks for identification of key pathways and regulatory elements, predictive modeling of protein levels from gene expression data [156], genome-scale metabolic modelling, and network-based integration [157], are increasingly employed to reconcile discrepancies and extract biologically meaningful insights. These integrated analyses can identify key metabolic pathways, regulatory nodes, and interspecies interactions critical for optimizing coculture strategies.

## 9. Advances in Genetic Engineering and Synthetic Biology for Fungal Strain Optimization

Comparative genomic and transcriptomic analyses between relatively distant species of Ascomycetes (e.g., *Aspergillus niger*, *Aspergillus nidulans*, and *Penicillium subrubescens* from the Eurotiomycetes, and *Trichoderma reesei* from the Sordariomycetes) as well as Basidiomycetes (e.g., *Phanerochaete chrysosporium* and *Dichomitus squalens*) have revealed notable differences between these taxa in terms of enzyme encoding genes. Basidiomycetes typically possess fewer genes encoding hemicellulolytic, pectinolytic, and starch-degrading CAZymes, but a similar number of cellulolytic enzyme-encoding genes as in the Eurotiomycetes. The number of AA9 LPMO genes, however, is generally significantly higher in Basidiomycetes when compared to Ascomycetes. Basidiomycetes also have multiple copies of GH45 and GH131 endoglucanase enzyme-encoding genes, while these genes are generally present in only a single copy in Ascomycetes (with *T. reesei* lacking GH131). Basidiomycetes also possess fewer xylanase genes, indicating a lower hemicellulose degradation capacity. CAZymes from the GH10 and CE16 families are more abundant in Basidiomycetes, whereas GH3, GH43, GH51, and GH54 families are more prevalent in Ascomycetes. Basidiomycetes also typically show less diversity in pectinase-related genes. PL enzymes, for example, which degrade pectin, are more diverse in Eurotiomycetes (particularly in *A. nidulans*) and less represented in Basidiomycetes [51].

The diverse adaptive and enzymatic strategies of *Ascomycota* and *Basidiomycota* offer great potential for synthetic biology applications focused on increasing the efficiency of biomass degradation. However, despite their potential, these fungi, especially macrofungi, have been relatively neglected in the development of synthetic biology tools compared to yeasts and prokaryotes. The development of genetic engineering approaches to increase enzyme production is nascent and limited to a few model and industrial fungi [158]. Challenging factors to consider in engineering these fungi include genome complexity, intricate metabolic and cellular processes, slow growth rates, low yields of genetic transformation, and the secretion of unwanted enzymes [159]. Advancements in CRISPR/Cas9, recombinant DNA technology, and AI-driven computational tools are essential for modifying or introducing genes that enhance the enzymatic capabilities of specific fungi. These technologies enable the selection and combination of compatible ascomycetes and basidiomycetes strains to maximize enzyme production and the synthesis of secondary metabolites. Additionally, they help improve yields in cocultures by promoting either competition or collaboration in environments with different types of lignocellulosic biomass [157,159]. This progress not only boosts the industrial efficiency of fungi but also opens up opportunities for designing customized fungal consortia that can degrade biomass more effectively in competitive environments.

Classical strain improvement methods have been widely used to improve strains, as they can be applied when knowledge about the genetic basis or biosynthetic pathways of the organisms of interest is limited. Traditional techniques involve plating on solid media, measuring colony size, and screening for chemical resistance. Other approaches include the selection of random mutants with the desired characteristics, screening based on knowledge of metabolic pathways, and the analysis of marker genes. These methods, however, face challenges such as slow mycelial growth, incompatibility with liquid culture, and the need for resources. More modern techniques, such as microplate assays and droplet microfluidics, allow for the efficient separation of candidate mutants [159,160]. Additional classical approaches also include random mutagenesis, which employs physical or chemical agents to induce mutations; adaptive evolution, which selects for mutants better adapted to specific conditions; protoplast fusion, which combines cells from different strains or species to produce hybrids with desirable traits; and genome shuffling, which involves several cycles of protoplast fusion and recombination to generate strains with diverse, potentially synergistic genetic combinations [160]. These methods can be applied in coculture in two ways: inducing mutagenesis in an isolated strain (for later use in coculture) or inducing mutagenesis during coculture. The advantage of inducing mutagenesis in isolation is the enhancement of unique characteristics in a particular fungus. For example, a mutant of *Trichoderma afroharzianum*, named MEA-12, was developed through mutagenesis using N-methyl-N’-nitro-N-nitrosoguanidine (MNNG), Ethyl Methanesulfonate (EMS), Atmospheric and Room Temperature Plasma (ARTP), and adaptive evolution under high sugar stress. The mutant produced more cellulolytic enzymes and exhibited tolerance to glucose catabolic repression compared to the parental strain [161]. Ref. [162] also studied the in vitro evolution of laccases from *Pleurotus ostreatus* using error-prone PCR (EP-PCR) and DNA shuffling to enhance enzyme activity and stability. Mutations improved enzyme stability by reducing subdomain flexibility and increased activity by enhancing access to the enzyme’s active site. In this case, DNA shuffling did not yield effective variants, while EP-PCR was highly effective. Additionally, *Penicillium oxalicum* underwent mixed mutagenesis and, when cocultured with *P. ostreatus*, exhibited high levels of xylanase and laccase activity [163]. Promise has also been observed using classical mutagenesis. Ref. [164], for example, conducted classical mutagenesis via heat treatment, UV radiation, and NTG (3-nitro, 5-methylguanidine) to improve the production of tannase and gallic acid in a coculture of *Aspergillus foetidus* and *Rhizopus oryzae*. Improved strains showed higher yields of tannase, protease, and amylase, as well as increased production of gallic acid over a shorter incubation time. Production efficiency was also observed for 13 generations, demonstrating genetic stability.

In addition to the modification of target genes, synthetic biology approaches can also be applied to control gene expression levels. Gene promoters, which control the level and timing of gene expression, are either constitutive or inducible. Although constitutive promoters provide stable expression, cell growth can be impaired if expression is too strong. Inducible promoters, on the other hand, can allow phase separation. In other words, it could allow microbial strains to first establish growth before activating the production of specific enzymes or metabolites [159,160]. In the case of coculture, this would avoid an excessive metabolic load during the initial growth phases, improving the viability of the strain. A number of approaches for promoter manipulation have been described for gene expression control. The recombinant *Penicillium verruculosum* strain B1-537 was engineered to express heterologous β-glucosidase from *Aspergillus niger* (AnBGL) and an AA9 LPMO from *Trichoderma reesei* (TrLPMO) under the inducible gla1 (glucoamylase GH15) gene promoter, resulting in enzyme preparations that significantly increased the hydrolysis of lignocellulose, with the combination of AnBGL and TrLPMO obtaining the highest glucose yield [165]. Synthetic inducible promoters, like the Tet-on system, enable controlled gene expression across species using doxycycline (Dox). In this system, the reverse transactivator (rtTA) binds DNA only in the presence of Dox, activating transcription. Ref. [165] optimized a Tet-on system for *Aspergillus niger*, demonstrating precise, tunable control over gene expression based on Dox concentration and gene copy number. Using luciferase as a reporter, they confirmed tight regulation, with no background expression and rapid induction, even at bioreactor scales. In the two cases mentioned above, different microorganisms had individually regulated promoters that allowed for the staggered or coordinated expression of proteins. The combination of different ways of regulating inducible promoters in a coculture could allow for a more dynamic control of enzyme expression, such as regulating the metabolic pathways so that the strains in the coculture do not compete excessively for the same substrate at the same time (e.g., one more active on lignin, the other more active on cellulose, mimicking the natural succession of microbial communities).

Bidirectional promoters allow the simultaneous expression of two genes, making them ideal for expressing genes of interest along with selection markers. The bidirectional Ph4h3 promoter from histone H4.1 and H3 genes was tested by [166] for its ability to drive the expression of two fluorescent reporter genes, mRFP1 (monomeric red fluorescent protein 1) and mCitrine. This system successfully enabled a one-locus expression of heterologous biosynthetic gene clusters from *Aspergillus brasiliensis* in *A. nidulans*, facilitating the biosynthesis of malformin compounds. Ref. [167] similarly identified and characterized a bidirectional promoter in *T. reesei* within the intergenic region between the *sor1* and *sor2* genes, which are involved in sorbicillinoid pigment production. This promoter effectively regulated the simultaneous expression of both genes, making it the first bidirectional promoter to be characterized in *T. reesei.* The strength of the promoter was quantified, and it was successfully utilized for the co-expression of two cellulase genes, demonstrating its potential for developing multi-gene expression systems in *T. reesei*. Bidirectional promoters can be a powerful tool for optimizing gene expression in coculture systems, for example, by being used to co-express genes for different enzymes involved in a multi-step biosynthetic or degradation pathway. This would allow the generation of high levels of upstream and downstream enzymes needed for efficient biotransformations in a coculture, thus increasing the overall efficiency of the system. The ability to control the expression of two genes simultaneously can be particularly useful for adjusting metabolic pathways in coculture systems. In cases where two different pathways have to be coordinated—such as when one organism produces a precursor and the other converts it into a final product—a bidirectional promoter could ensure balanced expression of the necessary enzymes in both organisms. This would avoid the need for complex genetic constructions or the introduction of multiple promoters into the same strain.

Transformation methods are crucial for introducing DNA into host cells to enable gene expression or genome modification. Common techniques include the following: (i) protoplast-mediated transformation (PMT), where the fungal cell wall is enzymatically removed to allow the insertion of exogenous DNA, facilitating genetic modification. While it can accommodate various types of DNA, optimized protocols are required for efficient use [159,160]. Robust PMT protocols have been developed for fungi such as *Colletotrichum falcatum* Cf671 [168], *Madurella mycetomatis* [169], and *Sporisorium scitamineum* [170]; (ii) *Agrobacterium tumefaciens*-mediated transformation (ATMT), where this method uses *A. tumefaciens* to transfer genes of interest into fungal cells, achieving stable, single-copy insertions. It is highly efficient for many fungal species but incompatible with episomal plasmids. The ATMT system utilizes a binary vector system, an *A. tumefaciens* strain with a virulence plasmid, and a suitable fungal receptor. Successful ATMT applications include fungi like *Aspergillus oryzae* [171]. Studies such as that by Yörük and Albayrak [172] showed that electroporation is efficient for genetic manipulation in *Fusarium* species, while [173] demonstrated its use in *Flammulina velutipes*. Electroporation has also been successfully used for *Piriformospora indica* [174]. Synthetic biology tools for fungi include Golden Gate, MoClo, and GoldenBraid. Golden Gate assembly uses type IIS restriction enzymes for seamless ligation of multiple DNA fragments. Ref. [175] developed versatile Golden Gate vectors for *Penicillium chrysogenum* and *Sordaria macrospora*, enabling gene tagging and deletion with EGFP, mRFP, and 3xFLAG tags, as well as vectors for deletion cassettes with resistance markers and FLP/FRT-based cassettes. A highly efficient metabolic pathway construction and genome integration system, called GoldenMOCS, has been developed for *A. niger* using a Golden Gate cloning approach and CRISPR/Cas9-mediated integration. The system allowed the rapid and flexible construction of metabolic pathway variants with high transformation and integration efficiency, using a split-pyrG selection marker for the direct selection of positive transformants [176].

Genome editing technologies like CRISPR/Cas9 (Clustered Regularly Interspaced Short Palindromic Repeats/Cas9) allow for precise and efficient gene modification. Challenges include controlling expression levels, reusing components, species-specific optimization (for example, the human-optimized version of Cas9 does not work on certain fungal species, such as *T. reesei*), transformation efficiency, limited selectable markers, and the process of selecting transformants for the desired genotype and phenotype remains laborious and time-consuming [177]. Several reviews cover the advances and challenges of CRISPR technology applied to filamentous fungi. Although it is not the scope of this article to address all these aspects, we highlight some studies with particularly interesting results, which may have relevant applications for coculture and engineering of filamentous fungi. Ref. [177] developed a CRISPR/Cas9 method for gene disruption in *Aspergillus oryzae* using in vitro transcribed gRNA (guide RNA) and a linear selectable marker gene cassette. Ref. [178] also engineered *A. oryzae* to overproduce nutraceutical ergothioneine and the flavor molecule heme using a recyclable CRISPR-Cas9 system with RNPs (ribonucleoprotein complexes), achieving high integration efficiency with minimized off-target effects. Ref. [179] similarly employed CRISPR/Cas9 to improve endoglucanase activity in *A. fumigatus* LMB-35Aa by integrating the eglA gene from *A. niger* into the pksP gene locus, enhancing activity by 40%. The work of [180] established an efficient CRISPR/Cas9-based genome editing system for *Penicillium subrubescens by* generating Δku70 mutants to enhance homologous recombination. *P. subrubescens* is a promising fungal cell factory due to its rich repertoire of CAZymes for plant biomass degradation, and the development of the Δku70 mutant allows for potential editing to create strains with improved production of specific enzymes for industrial applications. The study by [181] also established an efficient CRISPR/Cas9 genome editing protocol for *Rasamsonia emersonii*, enabling the deletion of the transcriptional repressor ACE1, which led to a substantial increase in cellulase and hemicellulase production. The modified ∆ACE1 strain showed superior enzymatic performance compared to commercial references, highlighting its potential for industrial biomass conversion. Finally, ref. [182] developed a platform for the hyperproduction of glucoamylase in the thermophilic fungus *Myceliophthora thermophila*, expanding the CRISPR-Cas12a genome editing capabilities and manipulating regulatory and secretion pathway genes, including components of the SREBP and UPR pathways. The resulting strain, MtGM12, showed a 35.6-fold increase in amylolytic enzyme production compared to the wild type.

The development of genetic tools and synthetic systems for filamentous fungi could open up new possibilities for coculture, allowing microbial interactions to be engineered in a more predictable and efficient way. The metabolic diversity of fungi is already a valuable resource, but the ability to genetically modulate their metabolic pathways and gene expression could turn coculture into an even more strategic approach for biotechnology. Tools such as CRISPR and modular DNA assembly make it possible to create strains that interact in an optimized way, whether to activate silent biosynthetic pathways, synergistically produce enzymes, or make better use of complex substrates. As we advance in adapting synthetic systems for fungi, coculture can evolve from an empirical process to an engineered system, where different organisms are combined to perform complementary functions, maximizing biomass conversion and the production of compounds of interest.

## 10. Consolidated Bioprocessing in the Context of Fungal Cocultures

Bioconversion of lignocellulosic biomass involves enzymatic hydrolysis followed by fermentation into further industrial products. While these bioconversion processes are generally conducted separately, consolidated bioprocessing (CBP) technologies enable complete enzyme secretion, hydrolysis, and fermentation, either by a single microorganism or a microbial consortium [183]. With energy requirements and waste products minimized, this approach for the employment of microbial enzymes in lignocellulose hydrolysis and fermentation is considered a potentially cost-effective single-step strategy for the efficient bioconversion of lignocellulosic biomass into valuable by-products and meets the requirements of the concept of white biotechnology. Given that CBP demands efficiency in biodegradation of lignocellulose and synthesis of desired industrial products, such properties in a single microorganism are difficult to encounter naturally, with genetic modification of native cellulolytic or non-cellulolytic microorganisms typically required [184,185,186]. Although the proof of concept of CBP with single microorganism chassis has been established, for example, with the filamentous fungus *Fusarium oxysporum*, which has been shown to possess enzymes for both hydrolysis of lignocellulose and fermentation of reducing sugars into ethanol [187], commercial application for increased productivity remains a challenge [188]. Microbial consortia, however, can carry out the complex process of CBP through cooperation between different microorganisms, combining the three steps of production of hydrolytic enzymes, the hydrolysis of lignocellulose, and microbial fermentation. In such an approach, the division of hydrolysis and biochemical production steps between microbial specialists is common. These systems are also more modular, allowing for the production of different chemicals without the need for major genetic modification. In addition, the simultaneous removal and utilization of reducing sugars can help maintain high and continuous lignocellulose-degrading enzyme activities, since the rate of lignocellulose hydrolysis is always the rate-limiting step in any CBP system. Therefore, the use of microbial consortia is preferable in CBP, as it reduces the metabolic load when enzyme and chemical production pathways are incorporated into a single strain.

Given the considerable potential for biodegradation of lignocellulosic biomass with hydrolytic enzymes produced from fungal genera, such as *Trichoderma*, *Penicillium*, and *Aspergillus* [188], numerous examples of consortia in CBP applications have been reported. Ref. [189], for example, reported a synthetic fungal consortium for the production of fumaric and lactic acids from cellulose and lignocellulosic biomass. In their study, *T. reesei* was employed for cellulolose degradation, *Rhizopus delemar* for fumaric acid production, and *Rhizopus oryzae* for lactic acid production. Temperature, aeration, and culture conditions were all considered for consortium compatibility, with the accumulation of organic acids during the bioconversion process and subsequent pH reduction identified as critical factors in maintaining enzyme activities. Ref. [190] also reported the development of a CBP for the conversion of cellulose into itaconic acid. By employing *Trichoderma reesei* RUT-C30 as a hypercellulolytic producer for the degradation of cellulose and a strain of *Ustilago maydis* modified to produce itaconic acid, the system successfully enabled the direct conversion of cellulose into itaconic acid without the need for externally added cellulases. Although the performance of the CBP was inferior to that achieved by simultaneous saccharification and fermentation (SSF), this proof of concept revealed the potential of these two organisms in microbial consortia for CBP applications with lignocellulosic biomass.

CBP holds considerable promise for reducing cost and improving efficiency in lignocellulosic biofuel production due to its one-step integration. However, its scalability is currently limited by the need for advanced metabolic engineering and robust microbial strains. SSF offers a good compromise and is more feasible at the industrial scale today, while SHF (separate hydrolysis and fermentation) remains valuable for process optimization and flexibility [191]. CBP offers the greatest potential in reducing costs and increasing efficiency, although it is not yet appropriate for large-scale deployment. SSF strikes a good balance between efficiency and cost and has gained traction in industrial settings. SHF, while flexible and well-established, is less efficient and cost-effective in comparison to SSF. As research advances, particularly in metabolic engineering and synthetic biology, CBP is expected to become more viable and could play a central role in the next generation of biofuel and bioproduct manufacturing processes.

Yeast surface display technology is utilized to engineer strains that present a repertoire of hydrolytic enzymes from diverse sources on the cell surface. When multiple cellulolytic enzymes are co-displayed in close spatial proximity on the yeast surface, the resulting assembly is referred to as a “mini-cellulosome.” This configuration often results in significantly higher specific activity compared to systems in which the same enzymes are secreted individually into the extracellular medium. Fungal co-cultivation systems, by contrast, exploit the natural division of labor and enzymatic synergy among different species, enabling the generation of a highly diverse enzyme pool. However, such systems can pose challenges related to process control, scalability, and maintaining interspecies balance during cultivation. In comparison, yeast surface display offers a streamlined, monoculture-based strategy with centralized genetic control and improved tolerance to industrial stressors such as ethanol. While yeast display systems are generally easier to scale and standardize, they are inherently limited by the number of enzymes that can be effectively displayed on the cell surface. Achieving functional synergy among the displayed enzymes may also require substantial genetic engineering efforts.

Fungal CBP systems face several critical biological and engineering challenges for commercialization. For instance, many filamentous fungi, such as *T. reesei*, exhibit low ethanol tolerance [164], limiting their viability and productivity in high-titer fermentations. Similarly, in co-cultures involving *Aspergillus niger*, glucose repression can downregulate the expression of cellulolytic enzymes, reducing overall saccharification efficiency. Multi-omics analyses often reveal disconnects between transcriptional activation of key enzymes and their translation or secretion, partly due to inefficient protein trafficking or degradation. Metabolomic data may also uncover byproduct accumulation (e.g., organic acids) that inhibits metabolic flux toward the desired product. To overcome these limitations, strain engineering approaches such as introducing ethanol-tolerant genes from yeasts (e.g., *Saccharomyces cerevisiae* ADH1 variants), enhancing stress response pathways, or modifying transcription factors that mediate carbon catabolite repression (e.g., CreA knockout) have shown promise. Process optimization strategies can also resolve performance issues: implementing fed-batch feeding to manage sugar mitigation concentrations, co-culture timing adjustments to balance growth dynamics, or oxygen control to shift metabolic flux toward ethanol production rather than biomass or acid byproducts. When integrated with multi-omics data, these solutions enable a more rational and adaptive design of robust fungal CBP systems [192].

## 11. Conclusions

Fungal coculture represents a transformative strategy for replicating and enhancing natural biomass degradation processes within industrial settings. By leveraging the complementary enzymatic capabilities of diverse fungal consortia, this approach facilitates efficient lignocellulose breakdown and enables the production of diverse bioproducts, from biofuels to high-value compounds. Despite its promise, several challenges continue to constrain the broader application of fungal coculture. Key limitations include competitive exclusion between strains, inhibition by secondary metabolites, and scalability constraints in industrial biorefinery systems. Competitive interactions can reduce enzymatic synergy, while toxic metabolites can inhibit fungal growth or enzyme activity. Furthermore, scaling coculture systems from laboratory to industrial scale is technically demanding, particularly due to the need to balance growth dynamics, adequate oxygenation, and metabolite exchange. Advances in genetic engineering, synthetic biology, and bioinformatics are already helping to overcome many of these bottlenecks. The integration of omics technologies has been instrumental in elucidating complex metabolic networks, thereby accelerating enzyme discovery and enhancing metabolite profiling. Future research in fungal coculture will likely prioritize the development of high-throughput screening (HTS) methodologies to efficiently identify synergistic fungal pairings. Recent work has demonstrated the efficacy of microplate-based HTS assays in isolating lignin-degrading consortia with enhanced enzymatic activity profiles [193]. Additionally, optimization of bioreactor configurations, including compartmentalized or modular systems, has shown promise for accommodating the distinct physiological needs of fungal cocultures and enhancing metabolite yields [194]. Furthermore, integrating machine learning approaches with multi-omics datasets can significantly advance the rational design of consortia. For instance, predictive models based on biosynthetic gene cluster data have been successfully used to infer secondary metabolite bioactivity and guide fungal pairing strategies [195]. Emerging trends include the application of synthetic biology tools such as modular cloning platforms to design synthetic fungal consortia based on tailored metabolic capacities [196]. The integration of fungal cocultures with other microbial systems, such as fungal coculture-bacterial consortia, has been successfully explored to enhance lignocellulolytic enzyme activity [197] and broaden potential bioproducts [198]. With a focus on such areas, research will likely overcome current limitations and fully harness the potential of cocultures in sustainable biotechnological applications.

## Figures and Tables

**Table 1:** Summary of Cazymes and their mode of action in plant cell wall degradation.

Compound	Type	Characteristics	Enzymes Involved
Cellulose	α-Cellulose, β-Cellulose, γ-Cellulose	Cellulose is a linear polymer of glucose units linked by β-1,4 glycosidic bonds. It has crystalline and amorphous regions, with hydroxyl groups in the amorphous regions being more reactive. Types of cellulose: α-cellulose: Long chains, insoluble in NaOH, high DP (600–1500). β-cellulose: Short chains, soluble in NaOH, moderate DP (15–90). γ-cellulose: Very short chains, DP < 15, mainly hemicellulose.	Endoglucanases (EGLs), cellobiohydrolases (CBHs), β-glucosidases (BGLs), and auxiliary enzymes like Lytic Polysaccharide Monooxygenases (LPMOs) and Cellobiose Dehydrogenases (CDHs)
Hemicellulose	Xylan, Mannan, Xyloglucan, Mixed-linkage glucan	Xylan: with a backbone of β-(1→4)-D-xylopyranose. It contains side chains. Xylan is categorized into glucuronoxylan, glucuronoarabinoxylan, and arabinoxylan. Mannan: Composed of D-mannose residues. Xyloglucan: Features a backbone of D-glucose residues linked by β-1,4 bonds, often substituted with α-D-xylose. MLGs: Linear polysaccharides with glucose monomers connected by β-1,4 bonds, interrupted by β-1,3 bonds.	Xylan: endo-β-1,4-xylanases, β-xylosidases, α-L-arabinofuranosidases, α-glucuronidases, esterases, β-xylobiohydrolase, exo-β-1,3-galactanase. Mannan: β-mannanase, β-mannosidase, α-galactosidase, β-glucosidase and acetyl mannan esterase. Xyloglucan: α-xylosidase, β-galactosidase, α-fucosidase, arabinofuranosidase, xyloglucanase and endo-β-1,4-glucanase. MLGs: Cellobiohydrolase, β-1,3-1,4-glucanase, β-1,4-glucana.
Pectin	Homogalacturonan, Rhamnogalacturonan I, Rhamnogalacturonan II, Xylogalacturonan	Homogalacturonan: A homopolymer of GalA linked by α-D-1,4 bonds, which may be methyl-esterified at the C-6 position. Rhamnogalacturonan I: Composed of a GalA backbone alternating with rhamnose (-α-1,4-D-GalA-α-1,2-L-Rha-), where rhamnose may be substituted by galactose, arabinose, or other sugars. Rhamnogalacturonan II: The most complex polymer, featuring a homogalacturonan backbone and over 13 different sugars in its branches. Xylogalacturonan: Homogalacturonan backbone with xylose side branches.	Pectinolytic enzymes include protopectinases, esterases, and depolymerases, comprising both hydrolases and lyases such as polygalacturonases and pectin lyases. These enzymes can be classified according to their mode of action: Endoenzymes: Endopolygalacturonase, endopectin lyase, and endopectate lyase; Exoenzymes: Exopoly galacturonase; Modifier: pectin esterase.
Lignin	p-hydroxyphenyl (H), guaiacyl (G), and syringyl (S)	Lignin is composed of monolignols (p-coumaryl alcohol, coniferyl alcohol, and sinapyl alcohol), forming H, G, and S units, respectively. S units have methoxy groups at the 5 positions, limiting certain bonds. Lignins include diverse C-O and C-C bonds (e.g., β-O-4, β-5, β-β). Low S unit content favors cross-links, creating dense, degradation-resistant networks. Composition varies by plant type: softwoods are rich in G lignin, hardwoods in GS lignin, and grasses in HGS lignin.	Laccases, Lignin peroxidases (LiPs), Manganese peroxidases (MnPs), Versatile peroxidases (VPs, and lignin-degrading auxiliary enzymes), O-demethylase, β-etherase, Auxiliary Activity (AA), and glucuronoyl esterases.

**Table 2:** Examples of fungal cocultures applied to lignocellulosic biomass degradation.

Fungal Coculture	Interaction	Substrate	Main Outcome Compared to Monoculture	Reference
*T. reesei* + *A. niger*	Synergistic	Wheat bran hydrolysate	Increased FPase and endoglucanase activities	[92]
*T. reesei* + *A. tubingensis/A. brasiliensis*	Synergistic	Pretreated wheat straw	Enhanced hydrolysis efficiency; better enzyme productivity	[93]
*T. reesei* + *A. brasiliensis*	Synergistic	Sugarcane bagasse	Enriched CAZyme profile; improved saccharification	[95]
*T. reesei* + *Cochliobolus heterostrophus*	Synergistic	Sugarcane bagasse	Enhanced hemicellulase production (xylanase, β-xylosidase)	[29]
*T. reesei* + *A. niger*	Synergistic	Sugarcane bagasse	Co-production of xylanase, cellulase, and β-glucosidase	[30]

## Data Availability

No new data were created or analyzed in this study. Data sharing is not applicable to this article.
